# Characterization of *Klebsiella pneumoniae* ST11 Isolates and Their Interactions with Lytic Phages

**DOI:** 10.3390/v11111080

**Published:** 2019-11-19

**Authors:** Demeng Tan, Yiyuan Zhang, Mengjun Cheng, Shuai Le, Jingmin Gu, Juan Bao, Jinhong Qin, Xiaokui Guo, Tongyu Zhu

**Affiliations:** 1 Shanghai Public Health Clinical Center, Fudan University, Shanghai 201508, Chinachengmengjun@shphc.org.cn (M.C.); leshuai@shphc.org.cn (S.L.); jingmin0629@163.com (J.G.); baojuan@shphc.org.cn (J.B.); jinhongqin@sjtu.edu.cn (J.Q.); xkguo@shsmu.edu.cn (X.G.); 2 Institutes of Medical Sciences, Shanghai Jiao Tong University, Shanghai 200025, China

**Keywords:** *Klebsiella*, phage therapy, phage-host interaction, antimicrobial resistance

## Abstract

The bacterial pathogen *Klebsiella pneumoniae* causes urinary tract infections in immunocompromised patients. Generally, the overuse of antibiotics contributes to the potential development and the spread of antibiotic resistance. In fact, certain strains of *K. pneumoniae* are becoming increasingly resistant to antibiotics, making infection by these strains more difficult to treat. The use of bacteriophages to control pathogens may offer a non-antibiotic-based approach to treat multidrug-resistant (MDR) infections. However, a detailed understanding of phage–host interactions is crucial in order to explore the potential success of phage-therapy for treatment. In this study, we investigated the molecular epidemiology of nine carbapenemase-producing *K. pneumoniae* isolates from a local hospital in Shanghai, China. All strain isolates belong to sequence type 11 (ST11) and harbor the *bla*_KPC-2_ gene. The S1-PFGE (S1 nuclease pulsed field gel electrophoresis) pattern of the isolates did not show any relationship to the multilocus sequence typing (MLST) profiles. In addition, we characterized phage 117 and phage 31 and assessed the potential application of phage therapy in treating *K. pneumoniae* infections in vitro. The results of morphological and genomic analyses suggested that both phages are affiliated to the T7 virus genus of the *Podoviridae* family. We also explored phage–host interactions during growth in both planktonic cells and biofilms. The phages’ heterogeneous lytic capacities against *K. pneumoniae* strains were demonstrated experimentally. Subsequent culture and urine experiments with phage 117 and host Kp36 initially demonstrated a strong lytic activity of the phages. However, rapid regrowth was observed following the initial lysis which suggests that phage resistant mutants were selected in the host populations. Additionally, a phage cocktail (117 + 31) was prepared and investigated for antimicrobial activity. In Luria Broth (LB) cultures, we observed that the cocktail showed significantly higher antimicrobial activity than phage 117 alone, but this was not observed in urine samples. Together, the results demonstrate the potential therapeutic value of phages in treating *K. pneumoniae* urinary tract infections.

## 1. Introduction

*Klebsiella pneumoniae* is a non-motile pathogenic Gram-negative bacterium responsible for nosocomial infections in the urinary tract, respiratory tract, and blood of immunocompromised individuals [1]. The persistence of the *K. pneumoniae* pathogen in nosocomial infections has been attributed to its ability to form virulence determinant capsular polysaccharides with increased tolerance to disinfectants and antibiotics [2]. Despite concern about the development and dispersal of antibiotic resistance in pathogen communities, polymyxin antibiotics, particularly colistin, have been widely used in antimicrobial prophylaxis as well as treatment of infections caused by carbapenem-resistant strains of *K. pneumoniae* [3]. However, over the past few decades, colistin resistance has emerged and become more common, with strains displaying decreased susceptibility to polymyxins [3]. This suggests that a rapid emergence of pan-drug-resistant (PDR) clones is occurring worldwide [4]. Indeed, several PDR isolates of *K. pneumoniae*, some of which have even caused outbreaks, have already been described [5]. Therefore, there is a great need for the development of alternative approaches to antibiotics for controlling and preventing *K. pneumoniae* infections.

One of the most common sources of predation pressure on bacteria comes from bacteriophage or bacterial viruse. Phages can determine the size, composition, structure, and development of microbial communities [6]. Phages are by far the most abundant and diverse microorganisms on earth, with total numbers estimated at more than 10^30^, generally outnumbering microbial cells by 10-fold in most natural environments [6]. Lytic or virulent phages hijack the host cellular machinery for their own replication, and the phage progenies are released till the lysis of the host; therefore, lytic phages can theoretically be harnessed to prevent bacterial infections. Due to their host specificity, phages only target specific bacterial species without affecting commensal bacteria (e.g., gut flora), thus minimizing the chances of opportunistic infections. Their strong lysis and specificity led phage therapy to become revitalized to control pathogenic bacterial infections in the post-antibiotic era [7]. 

Research in phage therapy has been growing significantly and consistently, focusing especially on a variety of the ESKAPE pathogens (*Enterococcus faecium*, *Staphylococcus aureus*, *K. pneumoniae*, *Acinetobacter baumannii*, *Pseudomonas aeruginosa*, and *Enterobacter* species) [8,9,10,11,12,13]. These studies have provided strong evidence that emphasizing lytic phage as an alternative to antibiotics could be successful at controlling the level of bacterial density, and in some cases, reducing mortality and morbidity in patients [11]. However, a sustainable phage therapy development in the treatment of *K. pneumoniae* requires isolation of potential lytic phages, in vitro characterization, phage cocktail preparation and purification, phage kinetics and in vivo studies, as well as mechanistic understanding of phage–host interaction both in vitro and in vivo, especially with regards to sophisticated bacteriophage resistant mechanisms at various stages of phage infection.

In this study, a collection of *K. pneumoniae* strains were characterized, along with two phages (phage 117 and phage 31), in order to ultimately evaluate their potential for controlling the pathogen. The molecular profiles and characteristics of *K. pneumoniae* were assessed to determine the prevalence of the carbapenem-resistant pathogen, test antibiotic susceptibility, and investigate microbiological characteristics, with the aim of establishing a collection of well-characterized bacterial isolates. Subsequent culture experiments with phage 117 and a phage cocktail (phage 117 + phage 31) initially demonstrated a strong bacteriolytic activity against strain Kp36. However, the rapid emergency of phage-resistant subpopulations following the initial lysis of susceptible populations indicates that potential defense strategies against phage predation develop and promote diversity in the host populations. Therefore, properly developed phage products that meet the requirement for quality and safety can be indispensable in controlling *Klebsiella* infections in various settings. 

## 2. Materials and Methods

### 2.1. Bacterial Strains and Propagation of Bacteriophages

A total of nine *K. pneumoniae* strains were isolated from two elderly patients (>60 years) with urinary tract infections (UTI) at Shanghai Public Health Clinical Center, Shanghai, China. Phage 117 and phage 31 used in this study were kindly provided by the Chen lab (Hualien Hospital, Taiwan). All of the strains were stored at –80 °C in Luria Broth (LB) medium (10 g tryptone, 5 g yeast extract, and 10 g NaCl per liter) (Sigma-Aldrich, St. Louis, MO, USA) with sterile glycerol (25%, *vol/vol*). The *bla*_KPC_ gene was detected by PCR and sequencing with *K. pneumoniae* carbapenemase (KPC) primers KPC-F (5′-TGTAAGTTACCGCGCTGAGG-3′) and KPC-R (5′-CCAGACGACGGCATAGTCAT-3′) [14]. 

Proliferation of phage 117 and phage 31 was performed by using the double-layer agar method [15]. To obtain high-titer phage stocks, 5 mL SM buffer (50 mM Tris-Cl, pH 7.5, 99 mM NaCl, 8 mM MgSO_4_, 0.01% gelatin, Sigma-Aldrich, St. Louis, MO, USA) was added to a fully lysed plate (90 × 15 mm), and the top agar (5 g/L) overlay was shredded with a sterilized inoculation loop. In order to release phage particles, the mixture was collected in a conical tube, stirred gently for at least 2 h at room temperature (RT) and then centrifuged at 12,000× *g* for 10 min at 4 °C. The supernatant was filtrated through 0.22 μm syringe filter (Millipore, Billerica, MA, USA), and the phage lysate was titrated and stored at 4 °C. 

### 2.2. Multi-Locus Sequence Typing (MLST) and S1-Pulsed Field Gel Electrophoresis (PFGE)

*K. pneumoniae* isolates representing diverse spatial and temporal dynamics were subsequently typed by MLST. MLST PCR with seven housekeeping genes—*gapA* (glyceraldehyde 3-phosphate dehydrogenase), *infB* (translation initiation factor 2), *mdh* (malate dehydrogenase), *pgi* (phosphoglucose isomerase), *phoE* (phosphorine E), *rpoB* (beta-subunit of RNA polymerase), and *tonB* (periplasmic energy transducer)—was performed on all nine isolates following the protocol described on the Pasteur MLST site (https://bigsdb.pasteur.fr/klebsiella/klebsiella.html) [16,17]. PCR product was sequenced, and the sequence was analyzed.

S1-PFGE analysis of *K. pneumoniae* isolates was performed as previously described [18]. Briefly, plugs were made using a mid-log bacterial cell suspension in Tris-EDTA (TE) buffer, and further treated with S1 nuclease (Thermo Fisher Scientific, Waltham, MA, USA). Switch times were ramped between 3 and 36 s for 18.5 h at 6 V/cm. The gels were stained with 1 mg/mL ethidium bromide (Sigma-Aldrich, St. Louis, Mo, USA ) in × TBE for 30 min in the dark and then washed with distilled water for 90 min to remove excess stains. Gel was then visualized with the ChemiDoc MP imaging system (Bio-Rad Laboratories, Hercules, CA, USA). 

### 2.3. Antimicrobial Susceptibility Testing

Antimicrobial susceptibility testing with VITEK2 compact system (bioMérieux, Lyon, France) was performed using an AST N281 card to determine whether the bacterial isolate is categorized as susceptible, intermediate or resistant to the drugs, according to the manufacturer’s instructions. Results were interpreted according to the interpretive standards of the Clinical and Laboratory Standards Institute and further validated using the disk diffusion method [19]. Imipenem belongs to carbapenem antimicrobial agents, which was used to treat a variety of serious bacterial infections. Carbapenem resistance was defined as resistance to imipenem (minimum inhibitory concentration (MIC) breakpoint of ≥16 µg/mL) or ertapenem (MIC breakpoint of ≥8 µg/mL), following the revision of *Enterobacteriaceae* MIC breakpoints. For both colistin and tigecycline, we use an MIC breakpoint of ≤2 µg/mL as susceptible. The antibiogram was routinely performed by the disk diffusion method according to Clinical and Laboratory Standards Institute guidelines. 

### 2.4. Plasmid Manipulation and Bacterial Conjugation

Direct transfer of *bla*_KPC_ resistance into *Escherichia coli* DH5α was attempted as reported [14]. In order to examine the potential for conjugational transfer of carbapenem resistance of representing strain Kp36, plasmids were extracted from bacterial isolate Kp36 using the GeneJET Plasmid Miniprep Kit (Thermo Fisher Scientific, Waltham, MA, USA) and transformed into *Escherichia coli* DH5α by heat shock (42 °C for 90 s). The transformants were selected on LB agar containing Tienam (20 μg/mL). 

The hypermucoviscous strain Kp36 and the apramycin-resistant *E. coli* DH5α were selected as the donor strain and the recipient for a conjugation assay, which was performed as previously described by Milton [20]. In brief, donor and recipient cells were mix at a ratio of 10:1 and spotted on LB at 37 °C overnight. The mixtures were collected and then plated on LB agar containing apramycin (100 μg/mL) and ampicillin (50 μg/mL). The *bla_KPC_* harboring plasmid transconjugants were verified by PCR and sequencing.

### 2.5. Phage–Host Ranges and Efficiency of Plating (EOP)

Phage host range is a key property for phage therapy. In order to determine the host spectrums of phage 117 and phage 31, spot test assay was performed against all of the *K. pneumoniae* strains. Aliquots of 2 µL phage lysate at a titer of ca. 10^9^ plaque-forming units (PFU)/mL were spotted on the surface of the *K. pneumoniae* lawn inoculated with 250 µL bacterial cultures in exponential phase. After incubation for 24 h at 37 °C, the lytic ability of phage 117 and phage 31 was assessed using the clarity of plaques, which was scored as transparent, opaque or no inhibition. 

Overnight cultures of Kp36 and Kp36-117R were diluted 1:1000 in LB broth (Sigma-Aldrich, St. Louis, MO, USA), and grown at 37 °C with shaking until OD_600_ reached 0.8. Cell-free spent medium was prepared by centrifugation of an aliquot of the Kp36 and Kp36-117R cultures at 12,000× *g* for 10 min at 4 °C followed by filtration of the supernatant (0.22 µm). The effects of cell-free spent media from phage host strains were tested against all of the *K. pneumoniae* strains as a control. 

Phage 117 was further characterized for a more thorough quantification for the outcome of productive infection, such as the efficiency of plating (EOP). The EOP of phage 117 on each phage-sensitive strain was determined by plaque assay as described previously [15]. The relative EOP was calculated as the ratio between the titer of the phage on a sensitive strain and the maximum titer observed. The plaque assay was performed three times for each phage–host interaction and relative EOP are reported as the mean of three replicates. 

### 2.6. Susceptibility of K. pneumoniae to Phage Infection at a Multiplicity of Infection (MOI) of 0.1 and Selection of Phage Resistant Mutants

Bacterial survival was determined following infection of Kp36 with phages as follows: strain Kp36 was grown to early exponential phase with an OD_600_ of 0.3 and infected with phage 117, phage 31, and phage 117 + 31 cocktail (MOI = 0.1) without shaking at 37 °C in LB broth (Sigma-Aldrich). This was done in triplicate 25-mL liquid LB cultures along with parallel control cultures without phage. The effect of phage-mediated host cell lysis was monitored by regular optical density measurements at 600 nm over the 8-h incubation. 

At the 8-h time point, single colonies were isolated from the culture (Kp36 + 117) on LB plates and subsequently purified by restreaking in order to determine changes in phage susceptibility in the host community during phage–host interactions, as described above. 

To isolate phage 117-resistant mutants from the other 8 strains (Kp28–Kp35), the same experiment described above was repeated. All the phage 117-resistant mutants were tested further for their antibiotic susceptibility by the VITEK2 compact system as described above. 

### 2.7. Effect of Phage 117 Addition on Biofilm Formation in K. pneumoniae Strains

Biofilm formation was monitored in cultures following the addition of phage 117 using methods previously described by Tan et al. with some modifications [21]. In summary, 13-mL polypropylene plastic tubes (Sangon, Shanghai, China) filled with 5 mL LB were inoculated with 50 µL of mid-log bacterial inoculum and 50 µL of phage stock (MOI of 0.1) and incubated without shaking at 37 °C for 10 days. Parallel control cultures without phages were kept as a control. For each experiment, crystal violet staining was used as a standard protocol for quantification of biofilm biomass production. After decanting the liquid, tubes were washed twice with 1× PBS (Sigma-Aldrich) to remove any planktonic cells. The bound biofilm cells were stained with 0.4% crystal violet solution (Sangon, Shanghai, China) for 15 min at RT, and excess crystal violet was removed by rinsing the tubes with tap water and drying for 10 min. The quantification of the attached cells was performed by adding 6 mL of 33% acetic acid (Sangon, Shanghai, China) as crystal violet solvent and leaving for 5 min to allow the stain to dissolve. The absorbance was measured at the OD_595_ of the eluate. 

### 2.8. Phage Stability and Killing Assays in Urine

To test phage stabilities, we did a human urine assay. Urine was collected from healthy volunteers, who signed the written informed consent to allow them to participate in the studies. Urine was centrifuged (8000× *g*, 10 min, 4 °C) and filtered (0.22 µm, Millipore, Billerica, MA, USA) to remove bacteria. Phage 117 was inoculated in triplicate 30-mL urine cultures at a final concentration of ~6 × 10^6^ PFU/mL. The stability of phage 117 in urine was monitored by plaque assay at 1-h intervals for 8 h without shaking at 37 °C. 

In order to demonstrate the potential for phage therapy as an effective treatment of UTIs, we conducted time-killing assays in urine. Bacteria exponentially grown at 37 °C, 200 rpm, in LB broth (Sigma-Aldrich) were diluted with fresh medium to obtain an OD_600_ of 0.3 (approximately 5 × 10^8^ colony-forming units (CFU)/mL). Aliquots of 100 µL bacterial strain Kp36 was inoculated in triplicate 30-mL urine cultures with phage 117, phage 31, and phage cocktail (117 + 31) at an MOI of 0.1. The bacterial titer (in triplicate) was determined at 1-h intervals for 8 h without shaking at 37 °C. 

### 2.9. Bacteriophage Genome Extraction, Sequencing, and Bioinformatics Analysis

Phage genomes were extracted using spin columns (QIAamp MinElute Viral Spin Kit, Qiagen, Hilden, Germany), according to the manufacturer’s instructions. Genomic DNA was sent to the Chinese National Human Genome Center for sequencing using an Illumina Hiseq paired-end platform with read quality evaluated. All reads were de novo assembled using velvet V1.2.03. Unlike protein coding genes, genes for transfer RNA (tRNA) are often incorrectly or inconsistently annotated, which makes comparative analysis based on tRNA genes difficult. Here we examined tRNA using tRNAscan-SE v1.3.1 (http://www.cbs.dtu.dk/services/RNAmmer/) to predict transfer RNA in full genome sequence. For prediction and analysis of gene starts in phage genome, the GeneMarkS phage program (http://topaz.gatech.edu/GeneMark/genemarks.cgi) was used. ORFs were further predicted and annotated manually for specific functions using BLASTP and the Rapid Annotations Subsystems Technology (RAST) (http://rast.nmpdr.org/). In addition, phage virulence factor analysis was conducted using a curated dataset of virulence factors through the Virulence Factor Database (VFDB) (http://www.mgc.ac.cn/VFs/), offering a quick access to virulence factors in bacterial pathogens. 

### 2.10. Bacteriophage and Bacterial Morphology by Transmission Electron Microscopy (TEM)

Phage morphology was characterized by transmission electron microscopy (TEM). An aliquot of 10 µL phage lysate was loaded onto a TEM formvar-carbon-coated copper grid (copper; size, 300 mesh) and allowed to adsorb for 20 min. The grids were then floated on a 10-µL spot of 2% sodium phosphotungstate (pH 7.4; 0.02 µm pore-size filters) for 2 min for negative staining, followed by removing excess staining with filter paper. Grids were rinsed with water (Sigma-Aldrich) and air dried. Grids were then observed under TEM (80 Kv, JEM-2100, JEOL, Japan). Negative capsule staining was performed as described above.

### 2.11. Nucleotide Sequence Accession Number

The nucleotide sequence data reported in the present study are listed in the GenBank nucleotide sequence database under accession numbers MN149903 (phage 117) and MN149904 (phage 31).

## 3. Results and Discussion

### 3.1. MLST Analysis

The MLST provides a valuable approach and offers unambiguous data for the epidemiology of *K. pneumoniae* isolates [16], and according to the *K. pneumoniae* MLST database (https://bigsdb.pasteur.fr/), all of the nine isolates of the KPC-producing *K. pneumoniae* strains showed the same sequence type (ST11, 3-3-1-1-1-1-4), based on the analysis of seven PCR-amplified housekeeping gene products (Table 1). Our results are in line with previous studies which showed that ST11 is the dominant clone of KPC-producing *K. pneumoniae* in China, and is closely related to ST258, only with a single-locus variant (*tonB*) [14,22,23]. ST11 was first found in France, and it has since been reported worldwide, including in North America, South America, and most countries in Europe [24]. Recently, an ST11 KPC-producing *K. pneumoniae* was also isolated in Korea, indicating ST11 may be another dominant clone of KPC-producing *K. pneumoniae* [25]. Therefore, we speculated that ST11 would be a good recipient for capturing disseminated KPC-encoding plasmids, making these particular isolates easily transmitted between patients [14,22]. Although this study was not regional and might not correctly reflect the actual distribution of extended-spectrum β-lactamases (ESBLs) in Shanghai, China, these results may still have some important epidemiological implications. 

### 3.2. Antimicrobial Susceptibility Testing

Among the nine isolates, all nine (100%) were positive for the string test and were identified as hypermucoviscous *K. pneumoniae* (HMKP). The MICs of antimicrobial agents tested against all the nine isolates were first determined with the VITEK 2 system (bioMérieux, Lyon, France), which identified resistance to ampicillin, ampicillin/sulbactam, piperacillin/tazobactam, cefazolin, cefotetan, ceftazidime, ceftriaxone, cefepime, aztreonam, ertapenem, imipenem, amikacin, gentamycin, tobramycin, ciprofloxacin, levofloxacin, nitrofurantoin, and trimethoprim/sulfamethoxazole (Table 1). Moreover, all isolates were susceptible to colistin (≤2 μg/mL) and tigecycline (≤2 μg/mL). These nine strains harboring a plasmid-encoded KPC confer resistance to all cephalosporins, monobactams, and carbapenems, hence demonstrating high levels of antibiotic resistance.

Previous studies have reported that, in terms of antimicrobial susceptibilities of cephalosporins and ciprofloxacin, *K. pneumoniae* isolates from urine were more resistant than isolates from blood [26]. Moreover, *Klebsiella*-mediated infections in the elderly universally had higher rates of resistance compared to those from adult patients [26]. Infection or colonization with PDR Gram-negative bacteria, whether patients exhibit chronic or acute illness, is associated with a substantial rate of mortality and morbidity. 

### 3.3. S1-PFGE and Plasmid Manipulation

**Figure 1:**
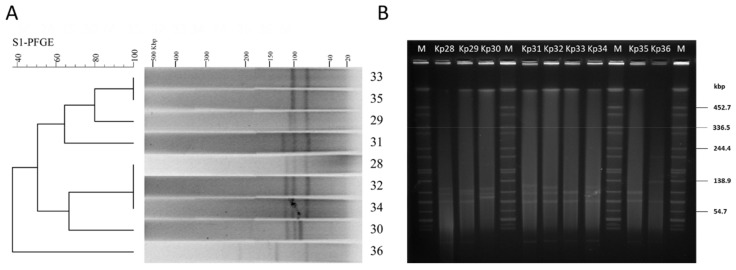
S1-pulsed-field gel electrophoresis analysis of isolated *K. pneumoniae* strains. (**A**) S1-pulsed-field gel electrophoresis fingerprint of the isolates; (**B**) molecular epidemiology investigation of the isolates; lane M: marker (*Salmonella* H9812); lane 28–36: Kp28–Kp36.

All of the nine isolates showed six different S1-PFGE types, containing multiple large plasmids (20~194 kb) (Figure 1A). The dendrogram and patterns of the S1-PFGE showed that four *K. pneumoniae* strain isolates (Kp33, Kp35, Kp29, and Kp31) had over 60% similarity, which grouped into the same PFGE cluster, while other strains (Kp28, Kp30, Kp32, Kp34, and Kp36) had less similarity (Figure 1A). UTI patient 1 colonized by a single MLST population of *K. pneumoniae* displayed a diverse population of plasmids with different sizes (Figure 1B). This confirms that various genotypes can emerge from a single patient with ST11 clonal infection. Further analysis of plasmid replicon typing is needed to facilitate the study of their roles in humans, as well as microbial adaptation and evolution.

*bla*_KPC_ and other resistance determinants are often located on plasmids [27], and S1-PFGE revealed that each of the isolated strains carries one or more plasmids (Figure 1A). In order to control the spread of multidrug-resistant (MDR) plasmid, we need to know many more variables that affect their movement. Transconjugation assays were performed using *E. coli* DH5α as the recipient. In this study, we failed to transfer *K. pneumoniae* isolates’ carbapenem resistance to *E. coli* DH5α by heat shock transformation, possibly because Miniprep kits may not be suitable for isolating plasmids of such a large size (Figure 1). However, conjugation assays showed that the ampicillin resistance determinant in strain Kp36 can be acquired by the recipient *E. coli* DH5α. Furthermore, the transconjugant DH5α-pKP36 was verified by PCR and subsequent sequencing and proved to be identical to that of strain Kp36. These results confirmed the presence of the *bla*_KPC_-harboring plasmid determinant, located on a conjugative or mobilizable plasmid in *K. pneumoniae* strain Kp36. 

### 3.4. Host Range and EOP

The host range and lytic potential of phage 117 and phage 31 were tested against nine KPC-producing *K. pneumoniae* strains by spotting 2 µL of phage lysate onto top agar containing different bacterial strains. The polyvalent phage 117 has a broad host spectrum and inhibits host growth to a great extent. Spot tests showed that phage 117 formed clear lytic zones on all the strains with an EOP of ca. 1. Turbid halos were formed around the lytic plaques of strains Kp32, Kp34, Kp35, and Kp36. However, phage 31 and cell-free spent culture medium of Kp36 and Kp36-117R showed no infectivity towards any of the strains. 

**Figure 2:**
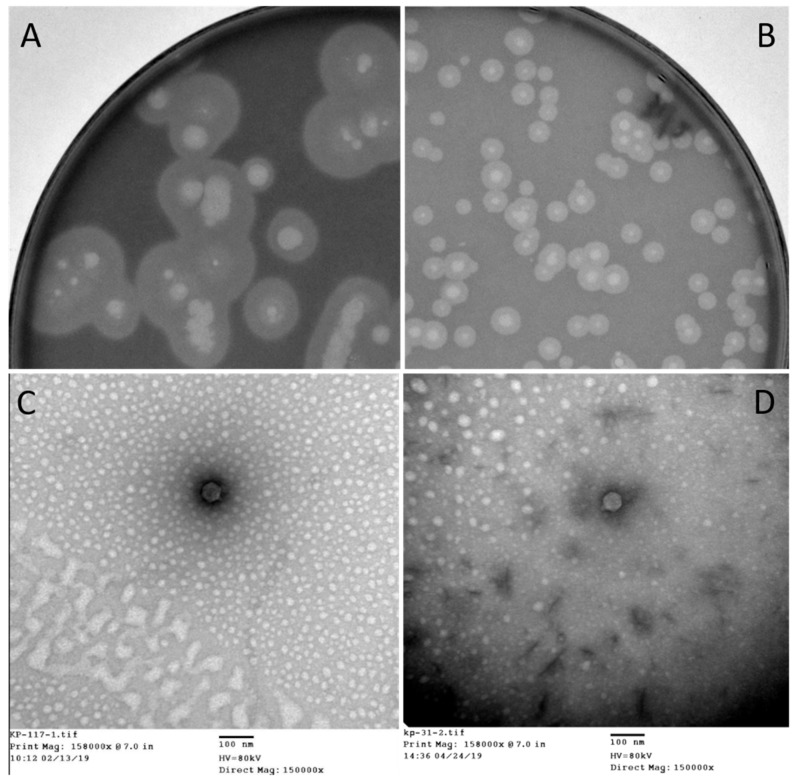
Plaque morphology images of (**A**) phage 117 and (**B**) 31. Plaques were observed in two distinct sizes. For (A) phage 117, the majority of plaques were ca. 0.8 cm in diameter; however, the plaques of (B) phage 31 were ca. 0.3 cm in diameter. The morphology of phage 117 plaques is consistently “halo” shaped. Transmission electron microscopy micrographs of bacteriophages: (**C**) phage 117 and (**D**) phage 31 classifying them to *Podoviridae* family. Scale bar, 100 nm.

The strain Kp36, an ST11 KPC-producing *K. pneumoniae* (KPC-Kp) which was isolated from patient 2, was used as a host to proliferate phage 117. The subsequent generations of phage 117 progenies were subjected to host range assay, and it corresponded to those of the first-generation assay. This indicates that phage 117 retained its broad host range when proliferated with host Kp36 alone. Phage 117 was detected as clear plaques with translucent halos (Figure 2A). Therefore, we hypothesized that phage 117 possesses capsule depolymerases, allowing it to recognize and digest specific capsular types. This further indicates that capsule depolymerase is crucial for successful infection of phage 117 in specific hosts. 

A bacterial capsule is largely composed of polysaccharides, which display multiple functions, such as adherence, resistance to immune clearance, protection against environmental factors, as well as phage receptors. During colonization in patients, the production of capsular polysaccharides is an essential virulence factor for *K. pneumoniae* in order to escape innate response mechanisms of the infected host, enabling the bacterium to establish infection successfully [28]. 

The bacterial cell wall plays key roles in phage–host adsorptions. Cell wall components (e.g., capsular polysaccharides, outer membrane protein, lipopolysaccharides, and flagella) can act as phage receptors in Gram-negative bacteria [29,30,31]. Bacteria often become resistant to phage infection by mutating or abolishing these exposed phage receptors. Interestingly, our results indicated that phage 31 can infect phage 117-resistant mutant Kp36-117R (Figure 2B), reflecting the diversity of infection mechanisms employed by phages in nature. In fact, other potential receptors for phage 31 targeting Kp36-117R likely exist, and this deserves further investigations. Similarly, in the case of phage SSU14 and phage SSU5, SSU14-resistant *Salmonella* mutants can be infected by phage SSU5 [32]. This mechanism is due to phage SSU5 recognizing O-antigens and utilizing the inner core of LPS as a phage receptor; therefore, it can eliminate O-Ag-specific phage SSU14-resistant mutants [32]. 

### 3.5. Phage–Host Interactions in LB Cultures and Urine Samples

The main aim of this study was to test the potential prophylactic and therapeutic application of lytic phages. Therefore, the antibacterial efficacies of the phage against drug-resistant bacterial pathogens in LB cultures and urine were the focus of the current study. The preliminary in vitro phage–host investigation above indicates that phage 117 had antibacterial activity against all the bacterial isolates. The logic of combining phage 117 and phage 31 stems from an evolutionary understanding that after strain Kp36 was exposed to phage 117, the phage 117-resistant mutant became susceptible to phage 31. Therefore, two sufficiently different selective pressures on the mixed populations (Kp36 and Kp36-117R) are likely to be more effective than either alone. We speculated that phage cocktail (117 + 31) could be a promising candidate for phage therapy to control *K. pneumoniae*, because of the ability to infect Kp36-117R mutants and thus delay the emergence of bacterial resistance. 

**Figure 3:**
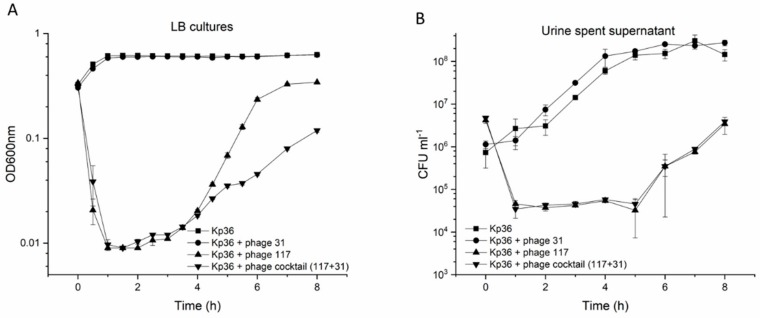
(**A**) Optical density (OD_600_) in cultures of *K. pneumoniae* amended with phage 117 (black triangle), phage 31 (black circle), and phage cocktail (117 + 31, black inverted triangle) at MOIs of 0 (control, black square) and 0.1 were measured at 1-h intervals over an 8 h period of incubation in Luria Broth (LB). (**B**) Lytic potential of phage 117 (black triangle), phage 31 (black circle), and phage cocktail (117 + 31, black inverted triangle) on strain Kp36 and control without phages (black square) at MOIs of 0.1. Cell proliferation and viability were determined at 1-h intervals post 8 h of incubation in cell-free urine spent supernatant. The corresponding concentrations of bacteria in cultures (CFU/mL) were measured by colony forming units as described in Section 2. Error bars represent standard deviations from all experiments carried out in triplicates (*n* = 3).

To verify this assumption, bacterial challenge tests were used to evaluate the activity of phages in vitro in liquid LB cultures. Phage 117 and the phage cocktail (117 + 31) were tested for their impact on the growth of hypervirulent Kp36 at an MOI of 0.1. Host growth in the test groups was significantly lower than that in the control group (*p* < 0.001). In the first 4 h, there was little difference between phage 117 and the phage cocktail (117 + 31) in host growth inhibition; the use of either phage 117 or the phage cocktail (117 + 31) delayed the onset of exponential growth in the same manner. However, a significant difference came after 4 h, and phage 117 and phage cocktail (117 + 31) reduced the maximum bacterial viability by >45% and >81%, respectively (Figure 3A). Both the growth rate and the final bacterial abundance were significantly decreased in the phage cocktail (117 + 31) groups. However, it should be noted that bacteria with different colony morphologies may result in differences in measured absorbance between wild-type and resistant mutants.

Next, we examined the influence of these urine components on the stability of phage 117 and phage 31 after exposure to the urine of a healthy volunteer. After 8 h of exposure to urine, there was no significant reduction in the concentration of infective phages of phage 117 and phage 31 (Figure S2). Moreover, based on the characteristics of the phages (phage 117 and phage 31), we designed a phage cocktail (117 + 31) and investigated its ability in controlling Kp36 cells at an MOI of 0.1 in urine. Both phage 117 and the phage cocktail (117 + 31) had strong control on the growth of strain Kp36 during the urine experiment, however bacterial abundance only showed a two-fold decrease (Figure 3B). Regrowth occurred after 5 h of incubation. In addition, phage-resistant mutants were isolated after 5 h with dramatic changes in morphology, turning from sticky to dry and smooth. The killing curves of the phage cocktail (phage 117 and phage 31) appeared to have no increased advantages in controlling bacteria growth, as shown in Figure 3B. 

In LB cultures, the cocktail (117 + 31) showed significantly higher antimicrobial activity than phage 117 alone, but this was not observed in urine samples. It may be possible to test whether the cocktail with different MOIs was effective in controlling growth of *Klebsiella* in urine, though it was not evaluated in this present study. Altogether, the findings of this study emphasize that in order to fulfill the potential of phage therapy, a more comprehensive understanding of in vitro and in vivo phage–host interactions in both LB cultures and urine is needed.

### 3.6. Effect of Phage 117 Selection on Bacterial Encapsulation

In addition to monitoring the bacterial abundance from the different phage conditions, we also obtained bacterial mutants during the incubations and further analyzed their changes in phage susceptibility. In the Kp36 + phage 117 and Kp36 + phage cocktail (117 + 31) cultures, the bacteriophage-resistant mutants were isolated after 8 h, after which colony morphology of Kp36-117 and Kp36-117-31 changed dramatically from extreme “stickiness” to dry and transparent. This suggests a cost of resistance in terms of reduced physiological capacity. Generally, there is a significant fitness cost of phage resistance mutations, and often it is associated with reducing abilities taking up specific nutrients or decreasing competitive abilities.

**Figure 4:**
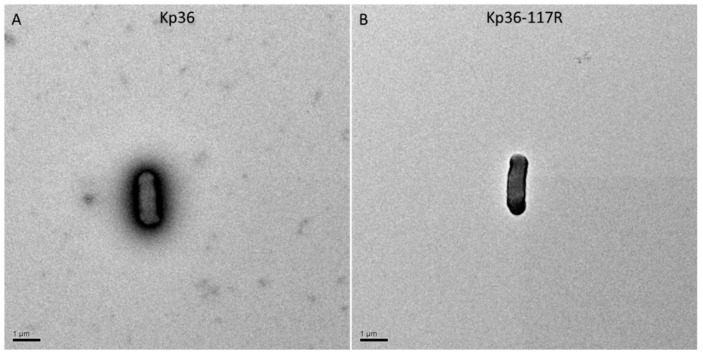
*K. pneumoniae* strains expressing different capsules were stained with phosphotungstic acid and were observed under a transmission electron microscope. (**A**) Kp36 (wild-type); (**B**) Kp36-117R (phage 117-resistant mutant). Scale bars, 1 µm.

Previously, studies have shown that phage-resistant colonies are rough, dry, and granulated, and phage-resistant mutants often lack many surface proteins, including capsules and lipopolysaccharides [33,34]. To examine this possibility, we employed transmission electron microscopy. There was a significant difference between strains Kp36 and Kp36-117R in the amount of fimbriae production observed at the surface of the capsulated cells, indicating that phage predation affects the synthesis or assembly of capsule expression (Figure 4). Other studies have found that deletion of glycosyltransferase gene (Δ*wcaJ*) can result in the significant decline of the lipid carrier undecaprenyl phosphate synthesis with the development of phage resistance and decline of virulence in the phage-resistant mutant, illustrating that WcaJ plays a major role in both maintaining phage sensitivity and bacterial pathogenicity [35,36]. In this study, the potential phage-resistant mechanism of Kp36 was not revealed. 

Results show that phage 117 selection on strain Kp31, Kp32, and Kp34 produces an evolutionary trade-off in KPC-producing *K. pneumoniae*, whereby phage 117-resistant mutants alter their antibiotic susceptibilities from resistant to intermediate/sensitive, causing increased sensitivity to several antibiotics (piperacillin/tazobactam, amikacin, gentamycin, tobramycin, ciprofloxacin, and levofloxacin) (Table 1). Our study highlights that rapid resistance evolution in phage–host interactions may influence antimicrobial susceptibility patterns. The exact underlying mechanism providing bacterial protection against phages, but increasing sensitivity to antibiotics has not been revealed in the present study. However, the fact that bacteriophages exert selection for MDR bacteria to become increasingly sensitive to traditional antibiotics represents a new approach to phage therapy. 

This combined effect is known as phage antibiotic synergy (PAS). Several studies have implied that PAS is common and can potentially affect phage–host interactions. Comeau et al. observed that sub-lethal levels of antibiotics can lead to a rapid process of phage maturation and accelerated cell lysis due to a change in bacterial cell morphology [37]. Also, the effect of exposure to sub-inhibitory concentrations of cefotaxime resulted in an observed increase in phage plaque size and phage production, which can significantly improve biofilm control in vitro [38]. Recently, *P. aeruginosa* phage OMKO1 selection leading to phage receptor (outer membrane porin M) mutation, which functions as the multidrug efflux systems MexAB and MexXY, caused increased sensitivity to drugs from several antibiotic classes [39]. The evidence that phage-resistant mutants become susceptible to antibiotics may potentially be used actively by combing antibiotics, hence preventing phage-resistant mutant subpopulation growth. In support of that, low-dose antibiotics in combination with phages demonstrated substantially increased survival rate over controls treated with phages or antibiotics alone, further suggesting that the combination of phage and antibiotics may be a promising strategy in future antimicrobial therapy [40]. 

### 3.7. Phage Morphology and Full Genome Sequence 

The morphology of phage 117 and phage 31 was examined by TEM. According to the International Committee on Taxonomy of Viruses (ICTV), phage 117 and phage 31 belong to the family *Podoviridae* in the order *Caudovirales* with short tails and icosahedral heads. Phage 117 has short tails measuring 10 nm long and 9 nm wide and a capsid measuring 55 nm long in diameter (Figure 2C). The phage 31 capsid is 57 nm long in diameter with short tails of 10 × 9 nm (Figure 2D). 

In order to properly explore the use of phage therapy for treating complicated infections, it is essential to characterize individual phages. Moreover, it is critical to ensure that phages selected for potential therapy do not have the capacity to facilitate horizontal gene transfer among resident microbiota. Therefore, we characterized the genome of phage 117 and phage 31. 

The genome of phage 117 measured 41,096 bp in size with a GC content of 52%. The annotation of the genome of phage 117 is summarized in Figure S1A. A total of 47 putative ORFs were identified in the genome, with 30 ORFs annotated as functional proteins which can be further categorized into four modules: (i) phage structure (capsid and scaffold protein, major capsid protein, portal protein, scaffold protein, head protein, tail protein, distal tail protein, tail tape measure protein, tail completion protein, major tail tube protein and tail chaperonin protein); (ii) host lysis (holin and endolysin); (iii) DNA packaging (terminase small subunit, terminase large subunit, and portal protein); (iv) DNA replication (DNA primase-helicase, endonuclease, MazG pyrophosphatase, recombinase, single-stranded DNA-binding protein, exonuclease, and DNA polymerase subunit), and other potential functions. 

Phage 31 has a 39,600 bp genome encoding 47 proteins (Figure S1B). A unique feature of the genome of phage 31 is that it encodes S-adenosyl-L-methionine hydrolase (SAM hydrolase), which catalyzes the hydrolysis of S-adenosyl-L-methionine, cleaving it to form L-homoserine and methylthioadenosine. This enzyme is encoded by phage T3, which infects and lyses *E. coli* cells. SAM hydrolase can degrade S-adenosylmethionine from the *E. coli* cell, thereby inhibiting a variety of SAM-related activities [41,42], however cleaving of SAM does not appear to be the primary strategy by which T3 counteracts host restriction endonucleases, since a previous study showed that one mutant of T3 lost the SAMase activity without losing the ability to overcome host restriction [43]. It is possible that phage 31 benefits from these metabolic enzymes by inhibiting the metabolism of the infected strain Kp36-117, which could, in turn, increase the number of phage progeny. 

Comparative genome analysis of phage 31 and the related phage 117, showed that these two are 67.54% identical over 66% of their genomes at the nucleotide level. One interesting site of variation between the two phages was seen in their predicted phage tail fiber proteins, which could explain host range differences between phage 117 and phage 31, although the mechanisms underlying interactions between binding proteins of the bacteriophage (phage) and receptors on the bacterial cell surface were not identified thus far.

### 3.8. Biofilm Formation

Most clinical carbapenem-resistant *K. pneumoniae* isolates were able to form distinct biofilms [44]. Biofilm formation is one of the important factors in many species of pathogenic bacteria [45]. Relative to planktonic bacteria, the antibiotic tolerance of mature bacterial biofilms is much higher, and bacteria in biofilms can resist phagocytosis and attenuate inflammation in vivo, making them very challenging to eliminate [46]. In the case of bacteria in the *Klebsiella* genus, it is believed that biofilm formation promotes persistence by evading the host innate immune response and resisting the effects of antimicrobials.

One of the big obstacles in the application of phage therapy to control pathogens in clinical is the emergence of phage-resistant or phage-tolerant subpopulations. Bacteria employ a wide range of anti-phage defense mechanisms, and drive functional and genetic diversification of the bacterial population on the basis of the rapid development of phage-resistant mutants, highlighting the need to understand how bacteria acquire phage resistance [47]. In addition to this, biofilm formation provides a potential physical refugee and chemical barrier for the bacteria to avoid the interaction between phage receptor and phage binding protein, and thus may challenge the use of phage therapy to successfully control pathogens [48]. In the face of these challenges, a deeper understanding of phage–biofilm interactions is therefore essential for assessing the potential application of phages in eradicating *K. pneumoniae*-biofilm mediated infection. 

**Figure 5:**
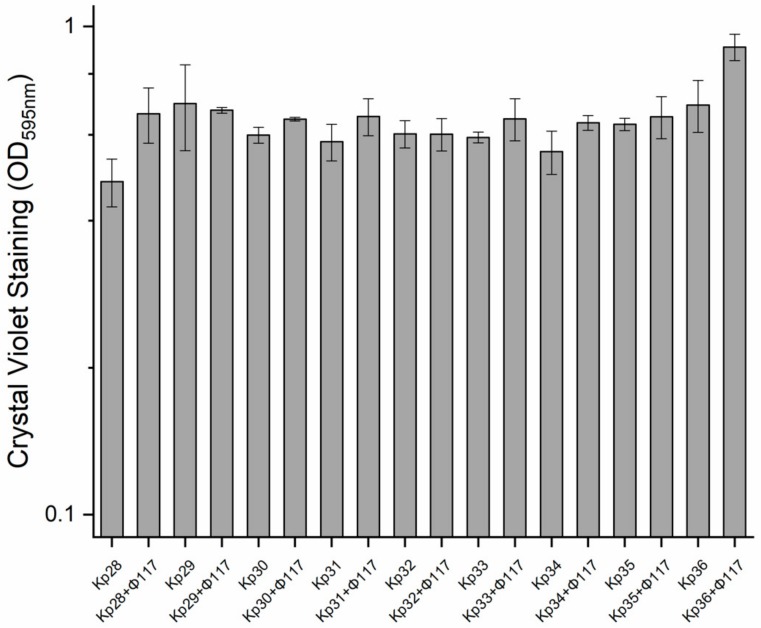
Biofilm formation of *K. pneumoniae* strains in cultures pretreated with phage 117, and control cultures, quantified by crystal violet (OD595nm, left y-axis). Error bars represent the actual ranges of data from all experiments carried out in triplicate.

In this study, the establishment and growth of biofilms produced by the nine *K. pneumoniae* strains in culture with and without phages (using only phage 117) were compared over the course of a long-term experiment. There were no significant differences detected among all the nine strains regarding their biofilm development (Figure 5). Interestingly, there was a slight increase in biofilm formation in cultures pre-treated with phage 117, relative to the controls (Figure 5).

However, it has been shown that different phages can affect their host bacterial biofilm formation differently in *Vibrio spp* and *Shewanella oneidensis* [21,49]. This was the case in a recent study that compared the effect of phage H20 (*Siphoviridae*) on the BA35 strain with the effect of phage KVP40 (*Myoviridae*) on the PF430-3 strain [21]. While the phage H20 inhibits biofilm formation in the host strain, phage KVP40 stimulates bacterial aggregates in the host strain at low cell density [21]. Current results showed that the resistant mutants increased biofilm production slightly, however, the difference was not statistically significant, suggesting a likely resistant mechanism to PF430-3. However, in the case of Kp36 and phage 117, enhanced biofilm formation may be due to genetic modifications and not to a quorum sensing-mediated phage receptor expression as it has been proposed for the PF430-3 strain [21,50].

A successful lytic phage life cycle depends on various factors including the ability of the phages’ binding protein to bind to the phage receptor of the susceptible bacterium and the host cell lysis. Polysaccharide depolymerases, which allow phage binding protein to specifically interact with phage receptors embedded in biofilm matrixes, have been observed in a variety of phages, in addition to this study [51,52,53,54]. Consequently, it is not yet known if the protection mechanisms observed in the Kp36 and phage 117 biofilm are widespread, because other phages may infect the Kp36 strain. 

## 4. Conclusions

In our present study, we characterized nine KPC isolates and two phages. Phage 117 and phage 31 have been fully characterized in vitro, and phage 117 could potentially be used as a biocontrol agent against *K. pneumoniae* ST11 isolates with carbapenem resistance. It can limit the growth of hypervirulent *K. pneumoniae* (hvKp) strain Kp36 in planktonic form and is relatively stable in urine. Clinical trials are required to judge the safeties and efficacies of potential therapeutic applications in vivo. Our results improve the current understanding of phage–host interactions in this important pathogen and further emphasize that phage–host interactions perhaps are more complicated than most laboratory studies and pose a challenge for the potential application of phage therapy.

The main limitation of this study was the small patient sample size which was taken from only one hospital in Shanghai. A large-scale investigation on the interactions between the lytic phages and carbapenem-resistant *K. pneumoniae* strains is in progress to provide strong evidence on the potential application of phage therapy to control these newly emerged strains in vitro and in vivo. Importantly, the increasing frequency of disseminated ST11 carbapenem-resistant hvKp in other regions of China highlights the issue of antibiotic resistance and their growing threat to human health, livestock, and the environment is becoming one of the leading problems [14]. Domestic surveillance and implementation of phage clinical trials is urgently required to prevent the dissemination of these novel strains in nosocomial settings. 

## Figures and Tables

**Table 1:** Multilocus sequence typing (MLST) type and antibiotic resistance characteristics of *K. pneumoniae* strains and their corresponding phage-resistant mutants. Abbreviations for antibacterial agents: AM, ampicillin; SAM, ampicillin sulbactam; TZP, piperacillin tazobactam; CFZ, cefazolin; CTT, cefotetan; CAZ, ceftazidime; CRO, ceftriaxone; FEP, cefepime; ATM, aztreonam; ETP, ertapenem; IPM, imipenem; AMK, amikacin; GEN, gentamycin; TOB, tobramycin; CIP, ciprofloxacin; LVX, levofloxacin; NIT, nitrofurantoin; SXT, trimethoprim sulfamethoxazole. Grey indicates phage 117-resistant mutant strains becoming more susceptible to antibiotics.

						Minimum Inhibitory Concentration (μg/mL)															
Strain Name	Source	*bla*_KPC_	MLST	AM	SAM	TZP	CFZ	CTT	CAZ	CRO	FEP	ATM	ETP	IPM	AMK	GEN	TOB	CIP	LVX	NIT	SXT
Kp28	Patient 1	Yes	ST11	≥32	≥32	≥128	≥64	≥64	≥64	≥64	≥64	≥64	>=8	≥16	≥64	≥16	≥16	≥4	≥8	≥512	≥320
Kp28-117R	Patient 1	Yes	ST11	≥32	≥32	≥128	≥64	≥64	≥64	≥64	≥64	≥64	≥8	≥16	≥64	≥16	≥16	≥4	≥8	≥512	≥320
Kp29	Patient 1	Yes	ST11	≥32	≥32	≥128	≥64	≥64	≥64	≥64	≥64	≥64	≥8	≥16	≥64	≥16	≥16	≥4	≥8	≥512	≥320
Kp29-117R	Patient 1	Yes	ST11	≥32	≥32	≥128	≥64	≥64	≥64	≥64	≥64	≥64	≥8	≥16	≥64	≥16	≥16	≥4	≥8	≥512	≥320
Kp30	Patient 1	Yes	ST11	≥32	≥32	≥128	≥64	≥64	≥64	≥64	≥64	≥64	≥8	≥16	≥64	≥16	≥16	≥4	≥8	≥512	≥320
Kp30-117R	Patient 1	Yes	ST11	≥32	≥32	≥128	≥64	≥64	≥64	≥64	≥64	≥64	≥8	≥16	≥64	≥16	≥16	≥4	≥8	≥512	≥320
Kp31	Patient 1	Yes	ST11	≥32	≥32	≥128	≥64	≥64	≥64	≥64	≥64	≥64	≥8	≥16	≥64	≥16	≥16	≥4	≥8	≥512	≥320
Kp31-117R	Patient 1	Yes	ST11	≥32	≥32	≥128	≥64	≥64	≥64	≥64	≥64	≥64	≥8	≥16	≥64	≤8	≥16	≥4	≥8	≥512	≥320
Kp32	Patient 1	Yes	ST11	≥32	≥32	≥128	≥64	≥64	≥64	≥64	≥64	≥64	≥8	≥16	≥64	≥16	≥16	≥4	≥8	≥512	≥320
Kp32-117R	Patient 1	Yes	ST11	≥32	≥32	≥128	≥64	≥64	≥64	≥64	≥64	≥64	≥8	≥16	≤32	≥16	≥16	≥4	≥8	≥512	≥320
Kp33	Patient 1	Yes	ST11	≥32	≥32	≥128	≥64	≥64	≥64	≥64	≥64	≥64	≥8	≥16	≥64	≥16	≥16	≥4	≥8	≥512	≥320
Kp33-117R	Patient 1	Yes	ST11	≥32	≥32	≥128	≥64	≥64	≥64	≥64	≥64	≥64	≥8	≥16	≥64	≥16	≥16	≥4	≥8	≥512	≥320
Kp34	Patient 1	Yes	ST11	≥32	≥32	≥128	≥64	≥64	≥64	≥64	≥64	≥64	≥8	≥16	≥64	≥16	≥16	≥4	≥8	≥512	≥320
Kp34-117R	Patient 1	Yes	ST11	≥32	≥32	≤16	≥64	≥64	≥64	≥64	≥64	≥64	≥8	≥16	≥64	≤4	≤2	≤2	≤4	≥512	≥320
Kp35	Patient 1	Yes	ST11	≥32	≥32	≥128	≥64	≥64	≥64	≥64	≥64	≥64	≥8	≥16	≥64	≥16	≥16	≥4	≥8	≥512	≥320
Kp35-117R	Patient 1	Yes	ST11	≥32	≥32	≥128	≥64	≥64	≥64	≥64	≥64	≥64	≥8	≥16	≥64	≥16	≥16	≥4	≥8	≥512	≥320
Kp36	Patient 2	Yes	ST11	≥32	≥32	≥128	≥64	≥64	≥64	≥64	≥64	≥64	≥8	≥16	≥64	≥16	≥16	≥4	≥8	≥512	≥320
Kp36-117R	Patient 2	Yes	ST11	≥32	≥32	≥128	≥64	≥64	≥64	≥64	≥64	≥64	≥8	≥16	≥64	≥16	≥16	≥4	≥8	≥512	≥320

## Supplementary Materials

The following are available online at https://www.mdpi.com/1999-4915/11/11/1080/s1, Figure S1: Genomic structure of phage 117 (A) and phage 31 (B). The genome map was performed using the CLC Main Workbench, version 12.0 (CLC bio, Qiagen, Denmark). Arrows represent predicted ORFs, the direction of the arrow represents the direction of transcription; Figure S2: Analysis of phage stabilities in the absence of host in cell-free urine spent supernatant. Culture was inoculated at 37 °C in a shaking incubator for 8 h. Error bars represent the actual ranges of data from all experiments carried out in triplicate.
