# Supplementary material for: Characterization of Klebsiella pneumoniae ST11 Isolates and Their Interactions with Lytic Phages
Source: Viruses. 2019 Nov 19;11(11):1080. doi: 10.3390/v11111080 (PMC6893751; doi:10.3390/v11111080)
Supplement: Supplementary file 1 [file viruses-11-01080-s001.zip › viruses-622901-SI.docx]

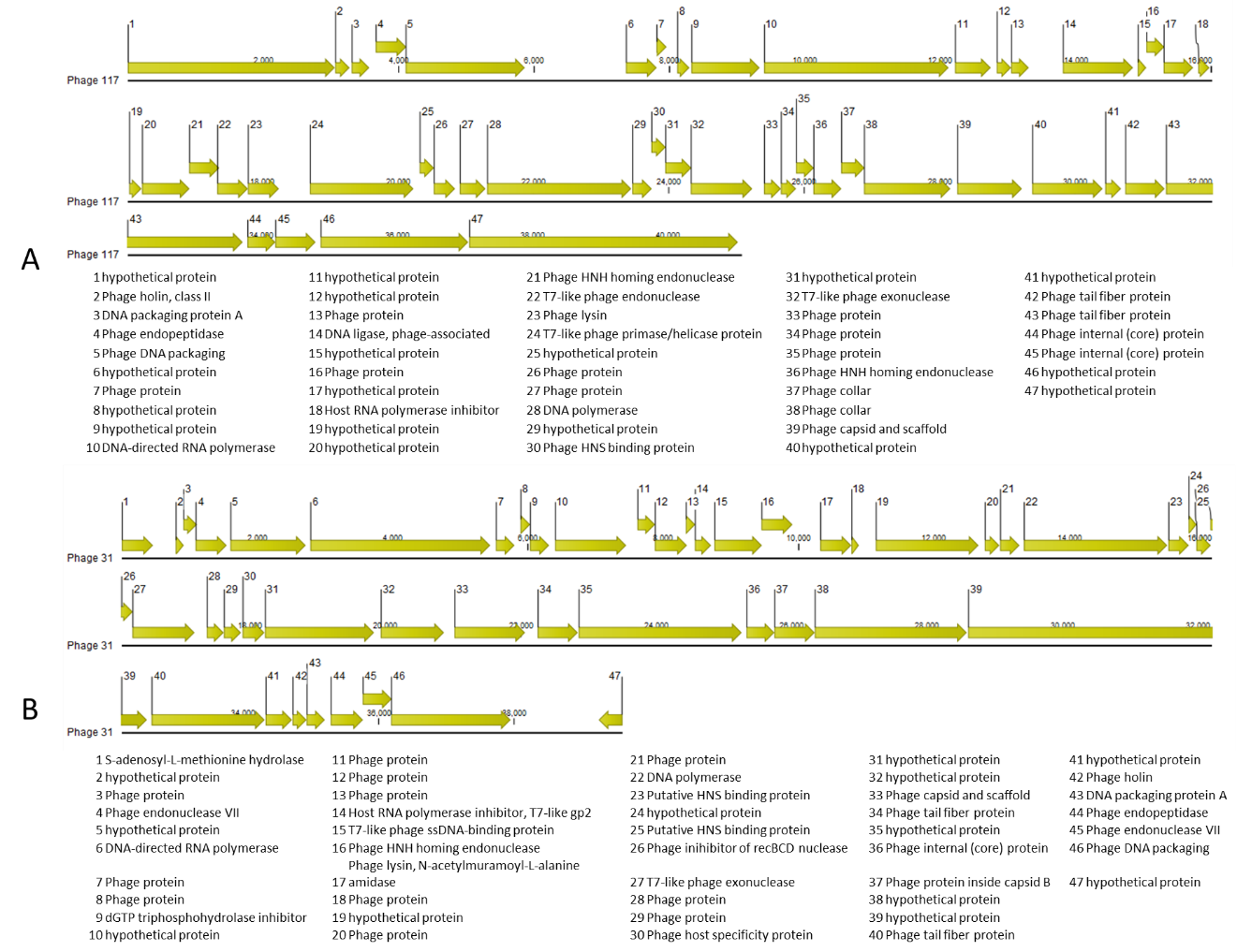


Figure S1: Genomic structure of phage 117 (A) and phage 31 (B). The genome map was performed using the CLC Main Workbench, version 12.0 (CLC bio, Qiagen, Denmark). Arrows represent predicted ORFs, the direction of the arrow represents the direction of transcription.





Figure S2: Analysis of phage stabilities in the absence of host in cell-free urine spent supernatant. Culture was inoculated at 37 °C in a shaking incubator for 8 h. Error bars represent the actual ranges of data from all experiments carried out in triplicate.
